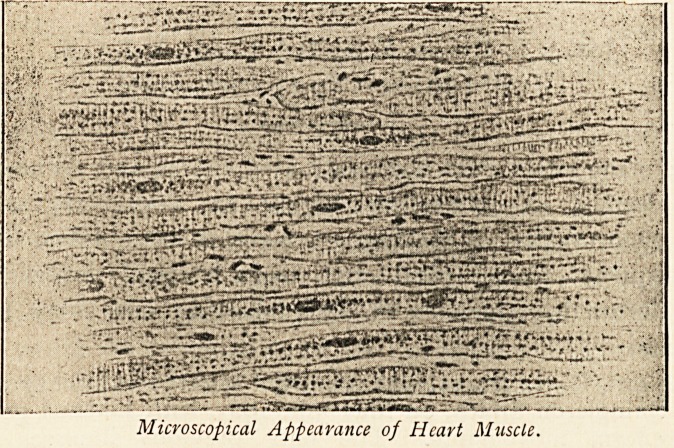# The Relationship of Chorea and Rheumatism

**Published:** 1904-09

**Authors:** Jos. J. S. Lucas

**Affiliations:** Assistant Pathologist, Royal Infirmary, Bristol


### THE RELATIONSHIP OF CHOREA AND RHEUMATISM.

BY

Jos. J. S. Lucas, M.B., B.A. Lond., M.R.C.S.,
*Assistant Pathologist, Royal Infirmary, Bristol.*

For many years past the relation of chorea and rheumatism has formed an interesting subject for discussion, and as the opportunities of making a *post-mortem* examination and a subsequent microscopical and bacteriological investigation are fortunately uncommon, the following case may be of interest.

A girl, C. A., æt. 14, was admitted into the Infirmary under Dr. Shingleton Smith, on May 5th, 1903, with marked general choreiform movements. The only previous illnesses were measles and whooping cough some years before, and she was apparently in good health until four days before admission.

Nothing noteworthy beyond the typical jactitations was found, as on examination the heart and lungs appeared to be normal. She gradually became worse and had to be removed to an isolation ward on May 10th, on account of her excitable condition. The temperature, which was 99° F. on admission, became of the intermittent type, rising to about 101° F. most evenings and falling practically to the normal in the mornings. This state continued for twelve days, and on May 22nd, after a meal of milk, she had marked dyspnœa and other signs of tracheal obstruction. Tracheotomy was performed, and she was temporarily relieved after a quantity of milk had exuded from the wound. Broncho-pneumonia, however, soon developed. The temperature rose the same evening to 104° F., and continued high until death on May 25th, two days afterwards.

*Autopsy* (about thirty hours after death).—The brain appeared to be normal. There were no signs of meningitis and no embolism, thrombus, nor hemorrhage could be discovered. The pericardium was healthy and the heart was rather larger than normal, weighing 9 oz. The mitral orifice was somewhat dilated, and the valve showed the usual appearances one sees in fatal cases of acute rheumatic endocarditis. Studded around the auricular surface close to the free edge were minute pedunculated vegetations forming a string of small pink beads. The heart muscle seemed healthy to the naked eye. The only other lesions discoverable were pleurisy at the right base and patches of broncho-pneumonia scattered throughout the right lung. This was the probable cause of death, and was evidently due to particles of inhaled food, some of which were found in the smaller bronchi.

*Microscopical Appearance of Heart Muscle.*

*Microscopical Examination.*—No further examination was made of the brain. Dr. Fisher examined sections of the heart muscle stained with Sudan iii. and hæmatoxylin, and the above illustration, which he has kindly lent me, is from one of these. He describes it[1] "as although in none the degeneration was extreme, every fibre in all the sections examined was dotted throughout with small granules of fat, the distribution being for the most part uniform, except that in many fibres the fat granules were most numerous in the neighbourhood of the ends of the muscle nuclei."

*Bacteriological Examination.*—Streak cultures upon Kanthack's medium were made from the inner surface of the pia-mater, the brain substance, the heart muscle, and the surface of the cardiac vegetations. On incubating the cultures from the pia-mater and brain, in twenty-four hours minute, discrete, clear, colourless round colonies appeared. These gradually coalesced, and in another forty-eight hours formed a thin greyish film on the surface of the medium. On examination of this growth it was found to consist entirely of small round diplococci tending to arrange themselves in chains, and staining well with ordinary reagents and with Gram's solution. Several broth tubes were at different times inoculated with the growth, but no change could be discovered after three days' incubation, and no diplococci could be found on microscopical examination. The original growth also soon died, and in about a week the film began to shrivel up and become opaque. The culture from the heart muscle gave negative results, but the one from the surface of the cardiac vegetations gave a copious growth of staphylococcus associated with a few colonies of a diplococcus similar in every respect to those found in the brain. Microscopical sections were also made through the diseased valve, and these, when stained by Gram's method, showed a few diplococci similar in appearance to those described above.

*Remarks.*—Briefly summarising the case, we have here a young girl, with no history of acute rheumatism, having chorea in a severe form, and showing *post-mortem* cardiac dilatation and mitral vegetations similar to those found in acute rheumatism. Also cultures made from the brain substance and pia-mater gave a pure growth of a diplococcus similar in most respects to Poynton and Paine's "rheumatic diplo-streptococcus."

As long ago as 1863 Kirkes expressed the opinion that chorea was due to cerebral irritation caused by separation of particles of vegetations of the mitral valves similar to those found in rheumatic fever, but a direct connection between rheumatism and chorea, I believe, was not suggested until 1894, when Sir Dyce Duckworth read a paper at the International Congress of Medicine at Rome[2] in support of the statement

[1] *Brit. M. J.*, 1903, ii. 452.

[2] *Atti d. xi. Cong. Med. Internaz.* (Rome), 1894, iii., Med. inter., p. 354.

that chorea was cerebral rheumatism. Since that time there has been a large amount of clinical and pathological evidence bearing on the subject. The frequent association of the two diseases in the same patient (60 per cent. of 115 cases recorded by Batten[1]), and the similarity of the cardiac and skin conditions, indicate very strongly that in many cases at least chorea is of rheumatic origin. *Post-mortem* evidence is naturally rather scanty, as cases of chorea seldom die; but such as it is, it points in the same direction. Various cerebral lesions have been described, such as multiple emboli (Kirkes) and minute hemorrhage and perivenous round-celled infiltration,[2] and these are suggestive at least of an infection such as rheumatic fever is now supposed to be. The condition of the heart is more conclusive. In the seventy-three autopsies collected by Osler[3] sixty-two had endocarditis, and most of these were of the simple variety only commonly seen otherwise in cases of rheumatic fever, and in the above case the minute vegetations on the mitral valves were typical of rheumatic endocarditis. Cheadle[4] goes so far as to say "that the endocarditis . . . of chorea" is "invariably rheumatic in nature and origin," and this appears to be the probable right conclusion.

As far as the bacteriology of chorea is concerned, ever since Mantle, at the British Medical Association in 1887,[5] propounded the idea that rheumatic fever is a bacteriological disease a large amount of work has been done. Various organisms (bacilli, staphylococci, streptococci, &c.) have been found both in rheumatic fever and chorea, and have been credited with the causation of the disease. The interest at present, however, centres round a diplococcus, or, as it is sometimes called, a diplo-streptococcus from its tendency to grow in chains. Dana in 1894[6] was the first to describe a diplococcus in a case of chorea following rheumatism, and since then a somewhat similar organism has been found in the blood, heart, and capsules of

[1] *Brit. M. J.*, 1903, ii. 450. [2] Reichardt, *Med. News*, 1902, p.
[3] Osler *Chorea and Choreiform Affections*, 1894.
[4] Allbutt's *System of Medicine*, vol. iii., 1897, p. 51.
[5] *Brit. M. J.*, 1887, i. 1381. [6] *Am. J. M. Sc.*, 1894, cvii. 31.

joints in rheumatic fever by Goldsheider, Michaelis, Litter, Wasserman, Triboulet, Coyon, Meyer, and others. Poynton and Paine have thoroughly investigated the subject, and a study of their recent publications[1] seems to make it pretty clear that a diplococcus is the causal factor in rheumatic fever. The evidence that chorea is produced by the diplococcus is, however, by no means so conclusive, as comparatively little work has been done. However, since Dana's experiment, Apert and Wasserman have found diplococci in the brain in fatal cases of chorea, and Poynton and Paine were able to find them in sections of a brain from a patient with chorea three years after death. These last observers also have produced choreiform movements in rabbits by injection of pure cultures of the diplococcus obtained from a case of acute rheumatism,[2] and more recently[3] Beattie has performed a similar experiment with a like result. Hence it may be said that bacteriological evidence, as far as it goes, strongly supports the idea that chorea is rheumatic in origin, and the presence of a diplococcus in the brain in the above case is some additional confirmatory evidence of this.

### HÆMATEMESIS ASSOCIATED WITH SMALL WHITE KIDNEYS.

[1] *Lancet*, 1900, ii. 861, 932. [2] *Tr. Path. Soc. Lond.*, 1901, lii. 248.

[3] *J. Path. and Bacteriol.*, 1904, ix. 276.

## Figures and Tables

**Figure f1:**